# Epidemiological Characteristics and Spatiotemporal Clustering of Pulmonary Tuberculosis Among Students in Southwest China From 2016 to 2022: Analysis of Population-Based Surveillance Data

**DOI:** 10.2196/64286

**Published:** 2024-09-24

**Authors:** Deliang Kong, Chengguo Wu, Yimin Cui, Jun Fan, Ting Zhang, Jiyuan Zhong, Chuan Pu

**Affiliations:** 1 School of Public Health, Chongqing Medical University, No. 1, Yixueyuan Road, Chongqing, 40016, China, 86 13320336327; 2 Institute of Tuberculosis Prevention and Treatment of Chongqing, Chongqing, China; 3 Qianjiang District Centre for Disease Control and Prevention, Chongqing, China; 4 Chongqing Institute of Tuberculosis Prevention and Treatment, Chongqing, China, +86 400050

**Keywords:** student PTB, Southwest China, epidemiology, visualizing incidence map, spatial autocorrelation analysis, spatiotemporal clusters, pulmonary tuberculosis

## Abstract

**Background:**

Pulmonary tuberculosis (PTB), as a respiratory infectious disease, poses significant risks of covert transmission and dissemination. The high aggregation and close contact among students in Chinese schools exacerbate the transmission risk of PTB outbreaks.

**Objective:**

This study investigated the epidemiological characteristics, geographic distribution, and spatiotemporal evolution of student PTB in Chongqing, Southwest China, aiming to delineate the incidence risks and clustering patterns of PTB among students.

**Methods:**

PTB case data from students monitored and reported in the Tuberculosis Information Management System within the China Information System for Disease Control and Prevention were used for this study. Descriptive analyses were conducted to characterize the epidemiological features of student PTB. Spatial trend surface analysis, global and local spatial autocorrelation analyses, and disease rate mapping were performed using ArcGIS 10.3. SaTScan 9.6 software was used to identify spatiotemporal clusters of PTB cases.

**Results:**

From 2016 to 2022, a total of 9920 student TB cases were reported in Chongqing, Southwest China, with an average incidence rate of 24.89/100,000. The incidence of student TB showed an initial increase followed by a decline, yet it remained relatively high. High school students (age: 13‐18 years; 6649/9920, 67.03%) and college students (age: ≥19 years; 2921/9920, 29.45%) accounted for the majority of student PTB cases. Patient identification primarily relied on passive detection, with a high proportion of delayed diagnosis and positive etiological results. COVID-19 prevention measures have had some impact on reducing incidence levels, but the primary factor appears to be the implementation of screening measures, which facilitated earlier case detection. Global spatial autocorrelation analysis indicated Moran *I* values of >0 for all years except 2018, ranging from 0.1908 to 0.4645 (all *P* values were <.05), suggesting strong positive spatial clustering of student PTB cases across Chongqing. Local spatial autocorrelation identified 7 high-high clusters, 13 low-low clusters, 5 high-low clusters, and 4 low-high clusters. High-high clusters were predominantly located in the southeast and northeast parts of Chongqing, consistent with spatial trend surface analysis and spatiotemporal clustering results. Spatiotemporal scan analysis revealed 4 statistically significant spatiotemporal clusters, with the most likely cluster in the southeast (relative risk [RR]=2.87, log likelihood ratio [LLR]=574.29, *P*<.001) and a secondary cluster in the northeast (RR=1.99, LLR=234.67, *P*<.001), indicating higher reported student TB cases and elevated risks of epidemic spread within these regions.

**Conclusions:**

Future efforts should comprehensively enhance prevention and control measures in high-risk areas of PTB in Chongqing to mitigate the incidence risk among students. Additionally, implementing proactive screening strategies and enhancing screening measures are crucial for early identification of student patients to prevent PTB outbreaks in schools.

## Introduction

Pulmonary tuberculosis (PTB) is a chronic respiratory infectious disease that poses a serious threat to human health and remains a significant global public health issue [1]. According to the “Global Tuberculosis Report 2023” by the World Health Organization (WHO), PTB continues to rank as the second leading cause of death from a single infectious agent, second only to COVID-19, with nearly twice as many deaths as HIV/AIDS, resulting in 1.3 million deaths globally in 2022 [2]. China is identified as one of the 30 countries with the highest burden of PTB, and despite a recent decline in PTB incidence, the situation remains severe due to its large population and high infection rates [3,4]. In 2022, China reported 748,000 new PTB cases, ranking third among these high-burden PTB countries [2].

PTB is characterized by a significant potential for latent transmission and high risk of dissemination. Without early diagnosis, timely treatment, and effective health management of patients with PTB, there can be proliferation of *Mycobacterium tuberculosis*, leading to the rapid spread of PTB epidemics in the population [5,6]. In China, the student population is substantial, with approximately 250 million enrolled students, which accounts for about 20% of the total population. Primary and secondary school students, who predominantly reside in boarding facilities, account for about 70% of this student body [7]. The prolonged gathering and close contact among students in classrooms and dormitories in China make schools highly vulnerable to PTB outbreaks and transmission [8,9]. Students have consistently been a primary focus of PTB prevention and control efforts, with China implementing measures such as physical examination of new students and reporting of absentee students to curb PTB incidence in schools [10,11]. However, given the rapid dissemination risks of PTB within school settings and its profound societal impacts, coupled with persistent reports of PTB outbreaks [12-14] and instances of multidrug-resistant PTB in Chinese schools [15-17], underscore the ongoing challenges in PTB prevention and control efforts within school.

Chongqing, situated in southwestern China, stands as one of the most significant and populous cities in the region and has historically been a high-incidence area for PTB in China. Previous reports indicate that from 2004 to 2021, the average incidence rate of PTB in Chongqing was 36.86/100,000, which was higher than the national average of 19.26/100,000 during the same period [18]. In 2020, Chongqing registered 1388 new cases of PTB among students, with an incidence rate of 24.96/100,000, surpassing the national student PTB incidence rate of 15.9/100,000 and ranking fourth nationwide [19]. Statistical data reveal that in 2022, Chongqing accommodated a total of 71 vocational and undergraduate institutions, enrolling 1.1057 million university students.Moreover, Chongqing is the sole city in China with over 2 million primary school students, totaling 2.0319 million students enrolled students [20]. The combination of high PTB incidence rates and extensive student enrollment places significant pressure on student PTB prevention and control efforts in Chongqing.

Geographic information systems and spatiotemporal clustering analysis are widely used in public health, particularly in the study of infectious diseases. These methodologies facilitate the identification of disease clusters, dynamic visualization of disease incidence and clustering trends across time and space, and the elucidation of geographic distribution patterns and risk levels in different regions [21-23]. In this study, we used spatial trend surface analysis, spatial autocorrelation analysis, and spatiotemporal clustering analysis methods to visualize the incidence and clustering of student PTB from 2016 to 2022 in Chongqing, Southwest China. This study aimed to investigate the epidemiological characteristics, geographical distribution, and spatiotemporal trends of student PTB in Chongqing, providing a foundation for clarifying PTB incidence risks among students and implementing precise and effective prevention and control strategies.

## Methods

### Study Area

**Figure 1.**
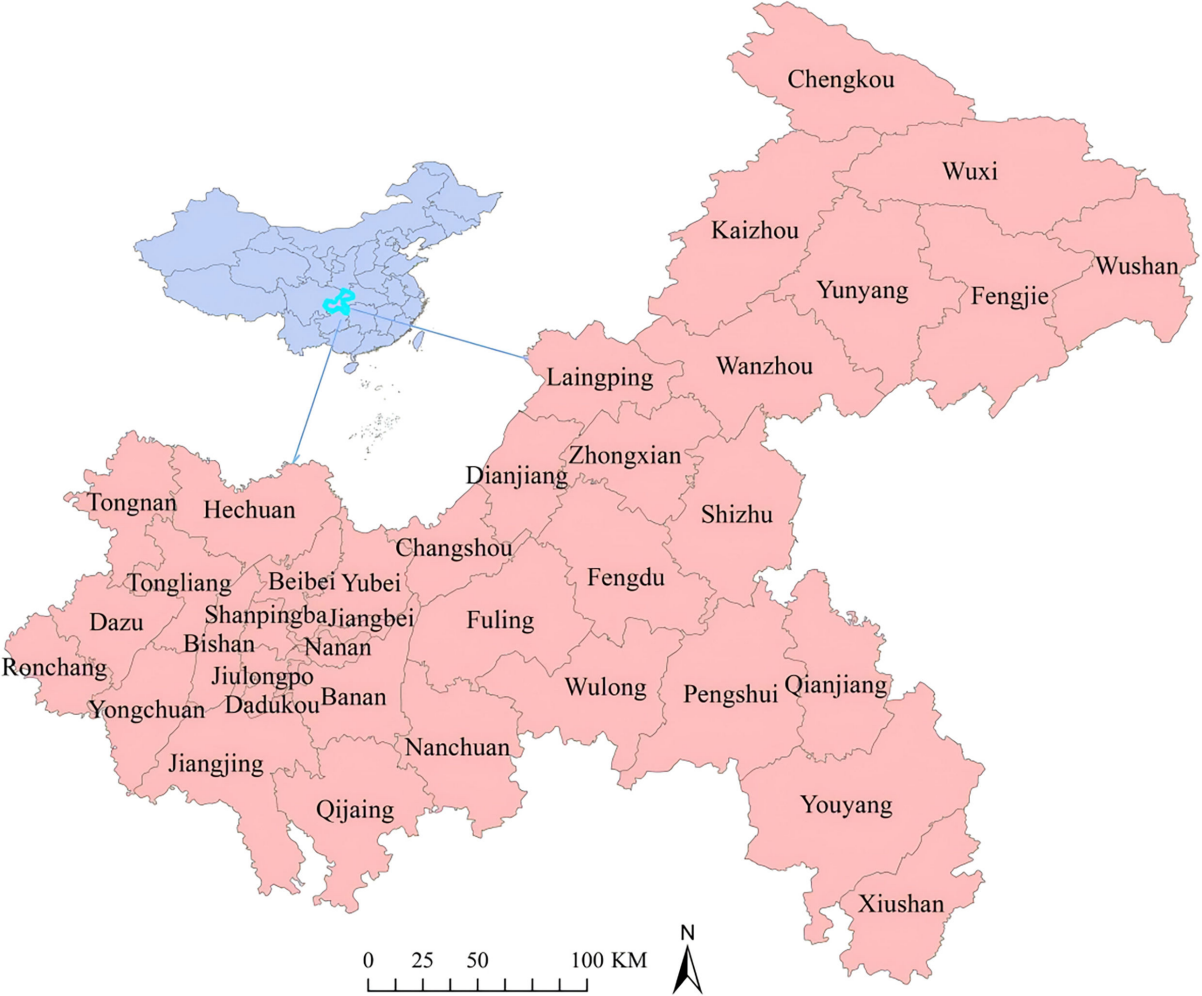
The geographical location and administrative division of Chongqing at the county level in China.

Chongqing is located between 28.10°N–32.13°N, 105.11°E–110.11°E, situated in the southwestern part of China and upstream of the Yangtze River. It is a direct-controlled municipality and serves as the economic center of the southwestern region. With an area of 82,403 square kilometers, Chongqing comprises 38 counties and had a permanent resident population of approximately 31.9143 million by the end of 2023. Chongqing features a subtropical monsoon humid climate, characterized by hot and humid summers and abundant annual precipitation. The average annual temperature ranges from 16 to 18 °C, with an average relative humidity of 70%-80% (Figure 1) [20].

### Data Collection and Management

PTB case data for Chongqing from 2016 to 2022 were sourced from the Tuberculosis Information Management System (TBIMS) within the China Information System for Disease Control and Prevention (CISDP). This system monitors and covers all PTB case reports from hospitals and public health institutions across Chinese provinces and municipalities. Definitions and classifications of PTB diagnosis follow the guidelines outlined in WS 288‐2017 PTB Diagnosis [24] and the Technical Guidelines for PTB Prevention and Control in China [25]. The etiological examination results for patients with PTB include positive, negative, and no results, with positive etiological results defined as any positive result from sputum smear, sputum culture, or molecular biological tests [24]. Each case within the TBIMS is allocated a unique medical record number to safeguard patient confidentiality and prevent redundant reporting, replacing personal identifiers such as names. The extracted information from student PTB medical records includes gender, age, ways of discovery, time of first symptoms, time of first consultation, initial health care facility, etiological results, treatment outcomes, and other related details. Reflecting the age groups and grades in China, students aged 3‐6 years, 7‐12 years, 13‐18 years, and ≥19 years are categorized as kindergarten students, primary school students, high school students (including junior and senior high school), and college students, respectively [7]. Population data for students in Chongqing’s counties from 2016 to 2022 were obtained from the “Chongqing Statistical Yearbook.” County-level vector maps of China and Chongqing were sourced from the National Basic Geographic Information System of China (Multimedia Appendix 1).

### Spatial Trend Surface Analysis

Spatial trend surface analysis is a statistical method used to analyze the geographical distribution and local variations of observed values (such as disease incidence rates) from a global perspective. It involves fitting a 2D polynomial regression model using linear models and mathematical surface fitting to study the distribution and changes in incidence rates at a regional scale [26]. The dependent variable Z (incidence rate) is modeled against the independent variables *X* and *Y* representing 2D coordinates. In this analysis, by projecting the latitude and longitude coordinates of different counties in Chongqing and their PTB incidence data onto a 3D scatter plot, this method aids in visualizing the incidence of PTB across different geographical locations and the spatial trends on a plane.

### Spatial Autocorrelation Analysis

Spatial autocorrelation analysis pertains to the degree of correlation between identical attribute values within adjacent spatial units [27]. Global and local spatial autocorrelation analyses were used to investigate the spatial clustering of student PTB cases. Global spatial autocorrelation analysis primarily uses the Moran *I* to describe the overall spatial distribution within the study area. The Moran *I* is computed after weighting and normalizing the spatial distance matrix, with values ranging from −1 to 1. A value of *I*>0 indicates positive spatial autocorrelation, *I*<0 indicates negative spatial autocorrelation, and values closer to absolute 1 signify stronger spatial correlation. *I*=0 denotes no spatial correlation, indicating a random distribution [28]. The calculation of the Moran *I* is outlined as follows:


$$I=\frac{n\sum_{i=1}^{n}\sum_{j=1}^{n}w_{ij}\left(x_{i}-\overset{´}{x}\right)\left(x_{j}-\overset{´}{x}\right)}{\sum_{i=1}^{n}\sum_{j=1}^{n}w_{ij}\sum_{i=1}^{n}{\left(x_{i}-\overset{´}{x}\right)}^{2}}$$

where n was the number of counties, $x_{i}$ and $x_{j}$ were the indicators of autocorrelations from unit index *i* and *j*, $\overset{´}{x}$ was average and $w_{ij}$ was the matrix of spatial weights. If unit *i* was adjoined to unit *j*, $w_{ij}$=1; if not, $w_{ij}$=0.

The Moran *I* is evaluated for significance using a *Z*-test, where *Z*>1.96 and *P*<.05 indicate statistically significant clustering of cases. The *Z*-test statistics can be expressed as:


$$Z=\frac{\left|I-E\left(I\right)\right|}{\sqrt{Var\left(I\right)}}$$

where E(I) represents the mathematically expected value of the Moran *I*; Var(I) was the variance of the Moran *I*.

Whether global spatial autocorrelation exists or not, local spatial autocorrelation analysis can uncover potential hot spots and local clustering patterns. Local spatial autocorrelation is described using the local Moran *I*, which characterizes specific clusters and their types [29]. The spatial distribution of these clusters can be visualized through the creation of local indicators of spatial autocorrelation (LISA) maps, presenting four cluster patterns: high-high, low-low, high-low, and low-high clusters [30]. The calculation method for the local Moran *I* is detailed as follows:


$$I_{i}=\frac{\left(x_{i}-\bar{x}\;\right)}{S^{2}}\sum_{j=1}^{n}W_{ij}\left(x_{j}-\bar{x}\right)$$

$$S^{2}=\frac{\sum_{j=1,j\neq i}^{n}W_{ij}{\left(x_{j}-\bar{x}\right)}^{2}}{n-1}$$

In the above formulas, $n,\;x_{i},\;x_{j},w_{ij}$, and $\bar{x}$ are the same as in the former formula.

### Spatiotemporal Clustering Analysis

The spatiotemporal scan analysis not only reveals the temporal trends of aggregated areas in geographical space but also assesses the relative risk (RR) of these clusters, providing a more accurate determination of spatial aggregation and risk levels. This analysis proceeds from both temporal and spatial dimensions by using cylindrical scanning windows over the study area, where the base represents the geographic region and the height denotes the time period [31]. Statistical inference is conducted through the calculation of the log likelihood ratio (LLR) and RR between observed and expected counts inside and outside dynamic windows of varying centers, radii, and time periods. A statistically significant difference in LLR values (*P*<.05) indicates spatiotemporal clustering, with higher LLR values signifying stronger clustering [32]. The cluster with the highest LLR value is identified as the most likely cluster, with additional statistically significant clusters ranked as secondary and 2nd secondary cluster. The formulas for LLR and RR are as follows:


$$LLR=log\left[{\left(\frac{c_{i}}{\mu_{i}}\right)}^{c_{i}}{\left(\frac{C-c_{i}}{C-\mu_{i}}\right)}^{C-c_{i}}\right]$$

$$RR=\left(\frac{c_{i}}{\mu_{i}}\right)\left(\frac{C-\mu_{i}}{C-c_{i}}\right)$$

where c represents the total number of cases, $c_{i}$ denotes the actual number of cases, and $\mu_{i}$ represents the expected number of cases. In this study, we used a space-time retrospective analysis using space-time scan statistics to analyze the distribution of student PTB cases in Chongqing from 2016 to 2022. The scanning period ranged from January 1, 2016, to December 31, 2022, with a basic time unit of 1 year and a basic geographical unit of administrative counties. The maximum temporal scanning window was set to 50% of the total study period, and the maximum spatial scanning area was set to 20% of the total population. Monte Carlo simulation was performed with 999 iterations to obtain *P* values, and clustering likelihoods of different windows were ranked based on the LLR value.

### Statistical Software

This study performed descriptive statistical analysis on the epidemiological and temporal characteristics of student PTB cases in Chongqing. Geographic information software ArcGIS 10.3 (version 10.3; Environmental Systems Research Institute, Inc) was used for visualizing incidence rate maps, spatial trend surface analysis, and global and local spatial autocorrelation analysis. SaTScan 9.6 software (version 9.6; Martin Kulldorff) was used for space-time scan analysis of student PTB case reports in Chongqing, exploring temporal ranges and spatial regions of student PTB clusters. Statistical significance was defined as *P*<.05.

### Ethical Considerations

The raw data used in this study were identified surveillance data from the China National TBIMS, and the surveillance data analyzed did not contain personal identifiers. This study was approved by the Ethics Committee of the Chongqing Tuberculosis Prevention and Control Center (202206).

## Results

### Epidemiological Characteristics

**Figure 2.**
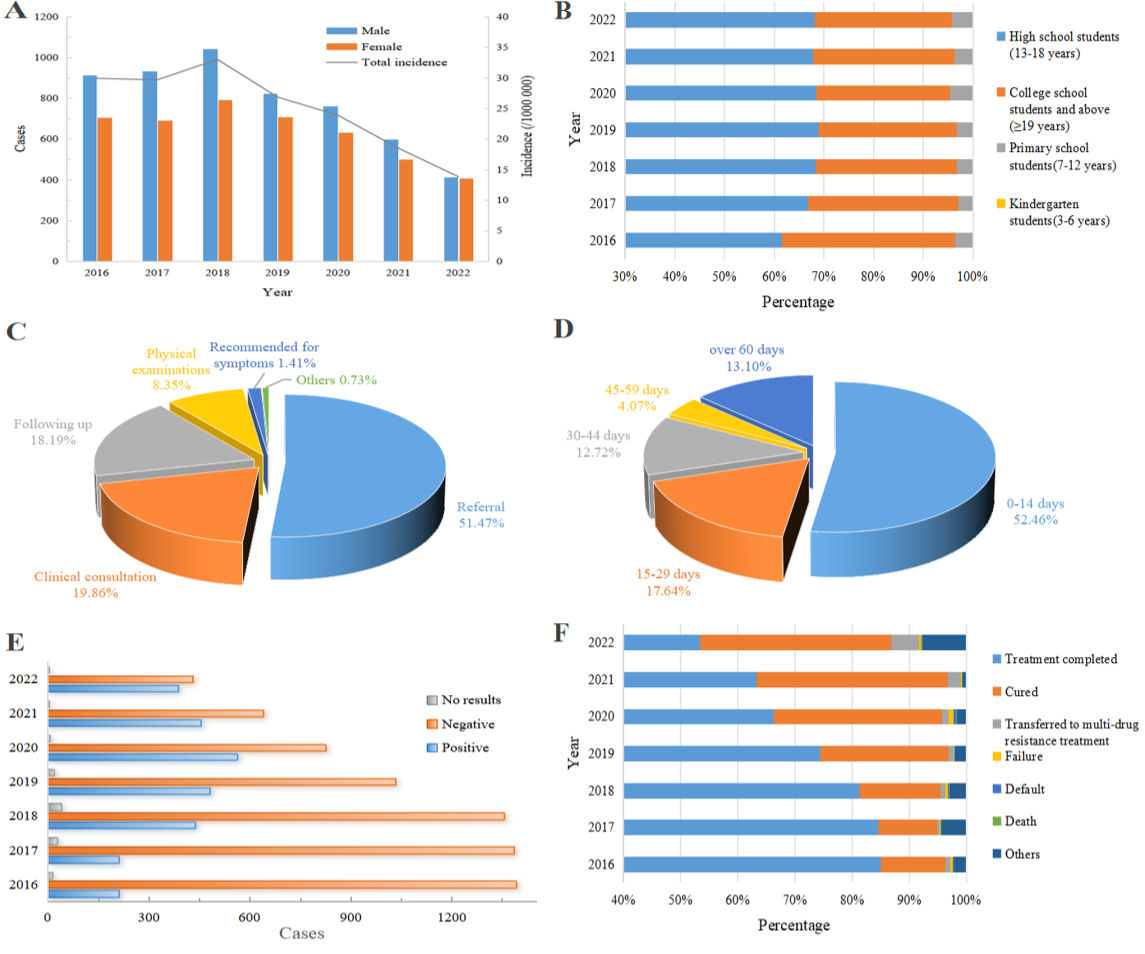
Epidemiological characteristics of pulmonary tuberculosis (PTB) among students in Chongqing, Southwest China, 2016‐2022. (A) The annual case number of PTB among students across different genders and the annual incidence of total cases. (B) The grade/age distribution. (C) Ways of discovery. (D) Interval between first symptoms occurrence to hospital visit. (E) Etiological results. (F) Treatment outcomes.

From 2016 to 2022, a total of 9920 student PTB cases were registered in Chongqing, Southwest China, with an average annual incidence rate of 24.89/100,000. The incidence of student PTB showed an initial increase followed by a subsequent decline during this period. The incidence peaked in 2018 at 33.00/100,000 and then steadily decreased to 13.82/100,000 by 2022, corresponding to an annual decline rate of 10.45%. Among all student PTB cases, males accounted for 55.28% (5484/9920) and females accounted for 44.72% (4436/9920), resulting in a male-to-female ratio of 1.24:1 (Figure 2A). In terms of grade or age distribution, the majority of cases were high school students (13‐18 years), comprising 67.03% (6649/9920), followed by college students (≥19 years) at 29.45% (2921/9920), and primary school students (7‐12 years) at 3.49% (346/9920) (Figure 2B). Referral was the primary method of case detection, accounting for 51.47% (5106/9920) of cases, followed by clinical consultation (1970/9920，19.86%), follow-up (1804/9920，18.19%), and physical examinations (828/9920, 8.35%) (Figure 2C). The interval between first symptom occurrence and hospital visit revealed that delayed presentation (more than 14 days) accounted for 47.54% (4716/9920) of cases (Figure 2D). Analysis of etiological results revealed a progressive increase in cases with positive etiological findings, rising from 13.11% (212/1617)in 2016 to 47.26% (388/821) in 2022 (Figure 2E). Treatment outcomes showed that the vast majority of patients (9446/9920, 95.22%) achieved successful treatment, including treatment completion and cure, with an increasing proportion of cured patients over time (Figure 2F).

### Temporal Characteristics

**Figure 3.**
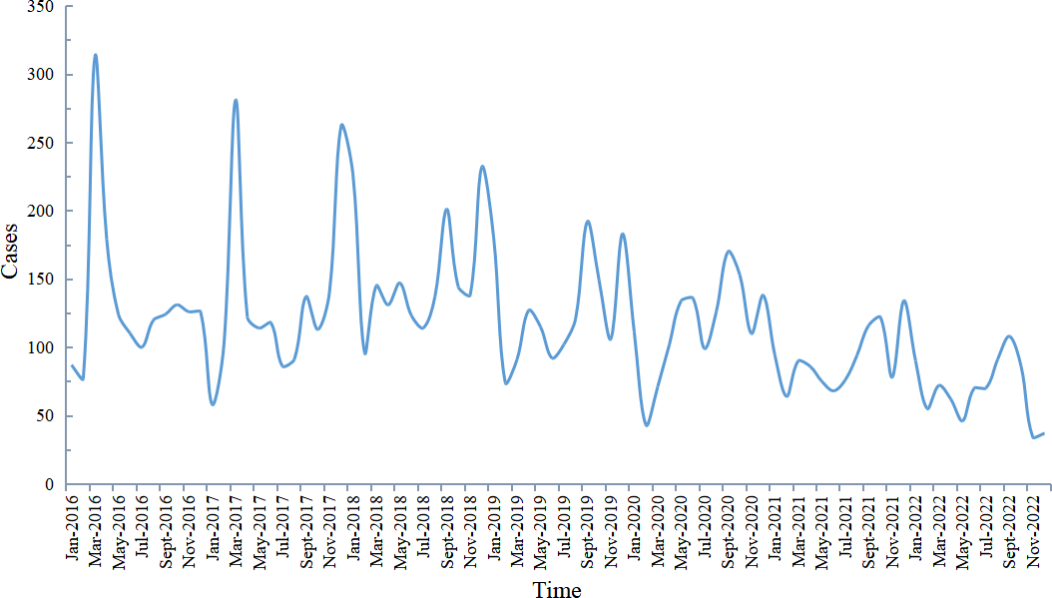
The monthly distribution of pulmonary tuberculosis among students in Chongqing, Southwest China, 2016‐2022.

Analysis of the monthly distribution of student PTB cases from 2016 to 2022 in Chongqing revealed distinct annual peaks, predominantly occurring in specific months each year (Figure 3). In 2016, the peak was in March, accounting for 19.36% (313/1617) of annual reported cases. In 2017, peaks occurred in March and December, representing 17.30% (281/1624) and 16.13% (262/1624) of the annual cases, respectively. From 2018 to 2022, a minor peak in March was noted each year, with major reporting peaks concentrated in September and December, with these months accounting for 15.69% (1048/6679) and 16.64% (1112/6679) of the total cases. Overall, student PTB cases in Chongqing exhibited a wave-like declining trend from 2016 to 2022.

### Spatial Characteristics

**Figure 4.**
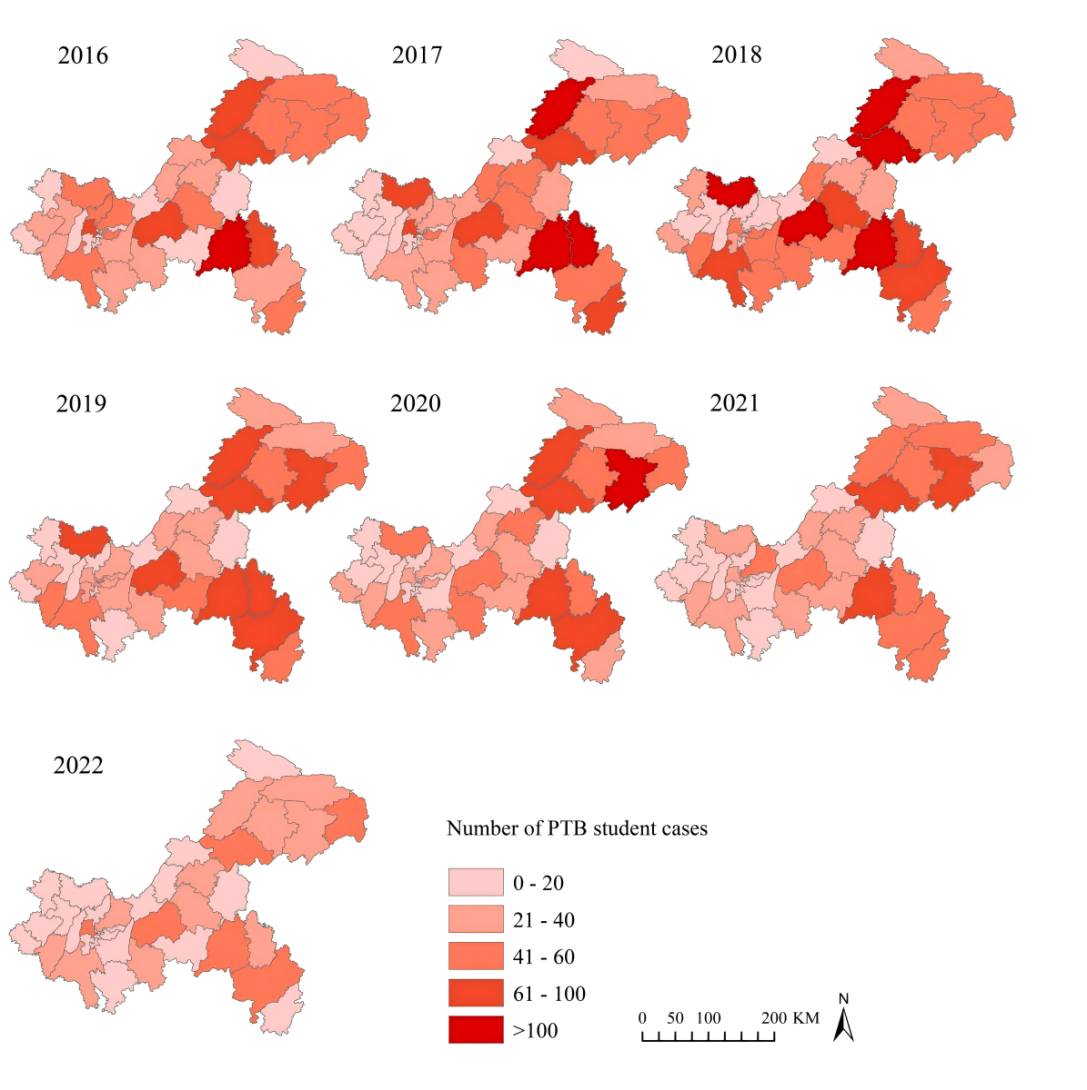
The spatial distribution of pulmonary tuberculosis among students in Chongqing, Southwest China, 2016‐2022.

There was significant variation in the number of reported student PTB cases between 2016 and 2022 across counties in Chongqing, with overall uneven distribution. High-incidence areas of student PTB remained primarily concentrated in the southeast and northeast parts of Chongqing. The top 5 counties reporting student PTB cases were Pengshui (660 cases), Kaizhou (622 cases), Wanzhou (561 cases), Qianjiang (549 cases), and Fengjie, (494 cases), all situated within these aforementioned high-incidence areas. From 2016 to 2018, there was a gradual increase in the area of dark-colored regions on maps, suggesting an upward trend in reported student PTB cases. However, after 2018, these dark-colored areas diminished, indicating a decline in reported cases during this period. As of 2022, 34 counties (89.47%) reported fewer than 40 student PTB cases (Figure 4).

### Spatial Trend Surface Analysis

**Figure 5.**
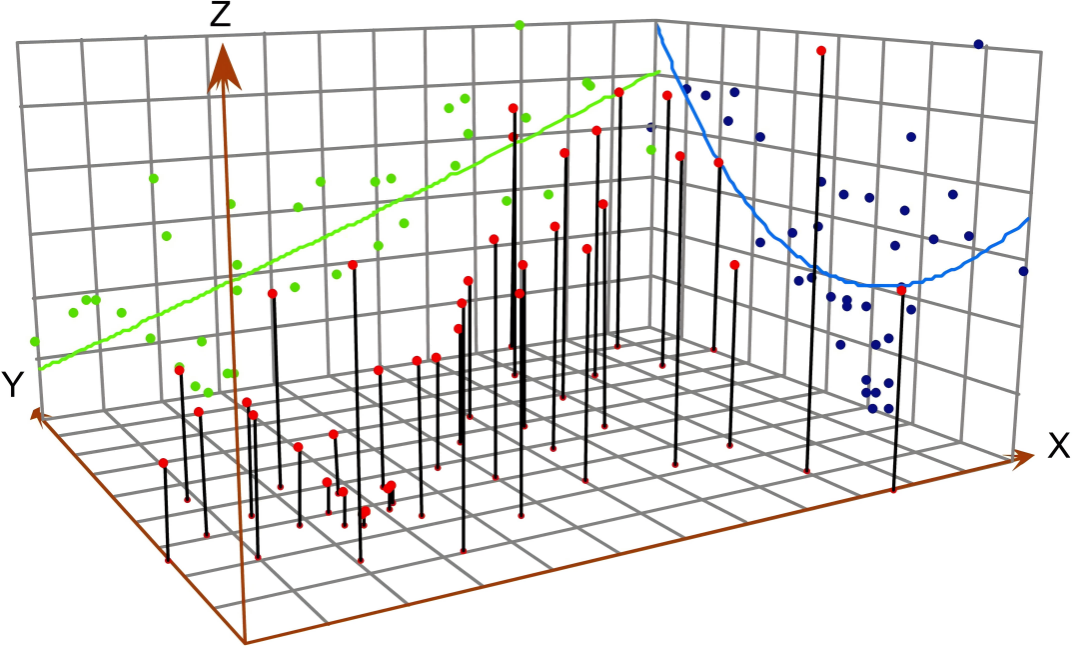
Spatial 3D trend surface analysis of pulmonary tuberculosis among students in Chongqing, Southwest China, 2016‐2022.

Using the number of student PTB cases reported from 2016 to 2022 across 38 counties in Chongqing as the dependent variable (z-axis) and latitude and longitude of regions as independent variables (x-axis and y-axis), a 3D trend surface map reveals significant spatial heterogeneity in student PTB incidence (Figure 5). The distribution of cases displayed a noticeable decreasing trend from west to east and a pattern of initial decrease followed by rapid increase from north to south in Chongqing from 2016 to 2022.

### Spatial Autocorrelation Analysis

**Figure 6.**
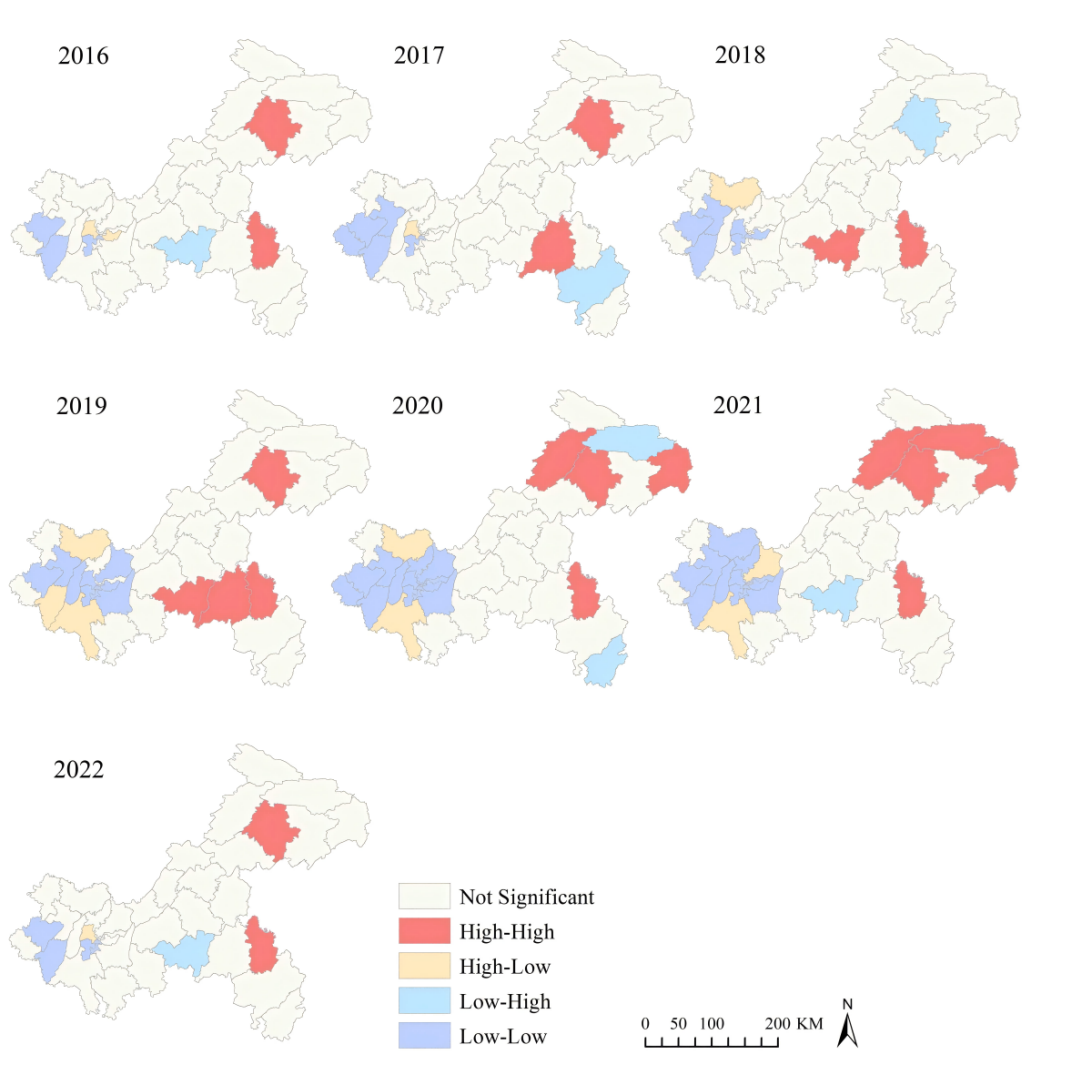
Annual local spatial autocorrelation of pulmonary tuberculosis among students in Chongqing, Southwest China, 2016‐2022.

Global spatial autocorrelation analysis of student PTB incidence across the 38 counties of Chongqing from 2016 to 2022 indicated that, except for 2018 where no significant spatial autocorrelation was observed (all *P* values were >.05), all other years showed global Moran *I* values of >0, ranging from 0.1908 to 0.4645 (all *P* values were <.05) (Table 1). This suggests a positive global spatial autocorrelation of student PTB incidence across the entire region. LISA maps for student PTB incidence identified a total of 7 high-high, 13 low-low, 5 high-low, and 4 low-high clusters throughout the study period (Figure 6). The high-high and low-low clusters were relatively stable in location annually, also indicating strong spatial clustering of student PTB cases. High-high clusters were predominantly located in the southeast and northeast parts of Chongqing, while low-low clusters were mainly observed in the central urban area and surrounding counties. Additionally, low-high clusters were detected around high-high clusters in most years except 2019, suggesting potential disease spread within the region.

**Table 1.** The global spatial autocorrelation of PTB among students in Chongqing, Southwest China, 2016‐2022.

Year	Moran *I*	*Z*-score	Mean (SD)	*P* value
2016	0.2200	2.3989	−0.0270 (0.0106)	.02
2017	0.2804	3.0562	−0.0270 (0.0101)	.002
2018	0.0652	0.9046	−0.0270 (0.0104)	.37
2019	0.3925	3.9856	−0.0270 (0.0111)	<.001
2020	0.4438	4.5586	−0.0270 (0.0107)	<.001
2021	0.4645	4.7261	−0.0270 (0.0108)	<.001
2022	0.1908	2.0546	−0.0270 (0.0112)	.04

### Spatiotemporal Cluster Analysis

**Figure 7.**
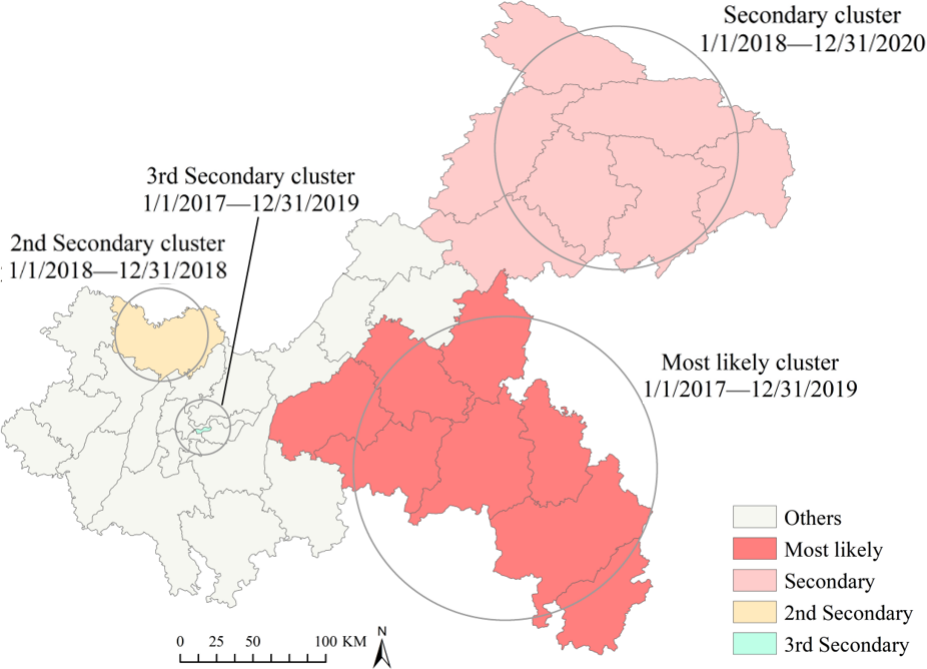
Spatiotemporal clusters of student pulmonary tuberculosis cases in Chongqing, Southwest China, 2016‐2022.

Spatiotemporal scan analysis of student PTB reported cases in Chongqing from 2016 to 2022 identified 4 significant clusters (Figure 7). The most likely cluster was located in the southeast of Chongqing, covering 8 counties with a cluster center at 28.8444 N, 108.7635 E, a radius of 164.34 km, and a clustering period from January 2017 to December 2019. Within this cluster, the student PTB incidence rate was 2.87 times higher than that in surrounding areas (RR=2.87, LLR=574.29, *P*＜.001). A secondary cluster was identified in the northeast of Chongqing, spanning 7 counties with a center at 31.0770 N, 109.8740 E, a radius of 150.45 km, and a clustering duration from January 2017 to December 2019. Here, the incidence rate of student PTB was 1.99 times higher than that in adjacent areas (RR=1.99, LLR=234.67, *P*＜.001). Additionally, statistically significant 2nd and 3rd secondary clusters were identified in smaller areas, including Hechuan in the north of the urban area (RR=2.76, LLR=57.49, *P*＜.001) and Yuzhong District in the main urban area (RR=1.75, LLR=17.77, *P*＜.001) (Table 2).

**Table 2.** The cluster results of the spatiotemporal scan for student pulmonary tuberculosis cases in Chongqing, Southwest China, 2016‐2022.

Cluster type	Most likely cluster	Secondary cluster	2nd secondary cluster	3rd secondary cluster
Counties, n	8	7	1	1
Cluster center	(28.844428 N, 108.763500 E)	(31.077043 N, 109.874000 E)	(29.975522 N, 106.273000 E)	(29.558923 N, 106.575354 E)
Radius (km)	164.34	150.45	0	0
Time frame	1/1/2017 to 12/31/2019	1/1/2018 to 12/31/2020	1/1/2018 to 12/31/2018	1/1/2017 to 12/31/2019
Population, n	4,541,765	5,491,176	1,258,377	591,846
Observed cases, n	1565	1360	154	138
Expected cases, n	607.59	733.91	56.44	79.51
Log likelihood ratio	574.28	234.67	57.49	17.77
Relative risk	2.87	1.99	2.76	1.75
*P* value	<.001	<.001	<.001	<.001

## Discussion

### Principal Findings

In this study, we explored the incidence trends, epidemiological characteristics, spatial distribution patterns, and epidemic aggregation risks of PTB among students in Chongqing using spatial trend surface analysis, spatial autocorrelation analysis, and spatiotemporal clustering analysis. These findings provide a basis for local health authorities to understand the epidemic trends of student PTB and implement targeted prevention and control measures. During the study period, the incidence of PTB among students in Chongqing exhibited an initial rise followed by a decline, with the prevalence increasing from 29.92/100,000 in 2016 to 33.00/100,000 in 2018, and subsequently decreasing consistently from 2018 to 2022, reaching 13.82/100,000 by the end of the study period. This trend aligns with the declining trend of PTB incidence in mainland China in recent years [33,34], suggesting a possible correlation with specific prevention and control measures targeting student PTB. In 2017, the Chinese Ministry of Education and Ministry of Health issued the “School PTB Prevention and Control Guidelines” [10], mandating health screening measures such as symptom screening, tuberculin skin testing, and chest radiography for new students. Chongqing subsequently integrated PTB screening into routine health exams for incoming students starting in 2018 [35]. Additionally, China integrated the school-based PTB case alert into the “National Infectious Disease Automatic Reporting System” in July 2018 [36], enhancing timely reporting of PTB cases among students.

After 2018, there was a significant decline in the incidence of student PTB in Chongqing. Considering the potential impact of the COVID-19 pandemic on respiratory infectious disease rates [37,38], we investigated the pandemic’s effect on the reported incidence of student PTB in Chongqing. The number of reported student PTB cases did not substantially increase after the pandemic, suggesting that the pandemic’s impact on delays or reductions in case diagnoses was minimal and did not significantly affect reporting levels [39]. Prior research show that PTB incidence rates in Chongqing’s general population [40] and among students [41] were decreasing before 2016. However, between 2016 and 2018, the incidence among students increased, contrary to the general population trend,likely due to the implementation of PTB screening measures. This suggests that the decline in incidence from 2019 to 2022 is likely attributable to increased screening rates, which promoted early detection and management of PTB among students. However, the COVID-19 preventive measures and changes in lifestyle, such as mask-wearing, reduced movement, school closures, symptom screening, heightened awareness of respiratory diseases, and improved personal hygiene, have indeed contributed to TB control among students [42]. Overall, the average incidence rate of PTB among students in Chongqing from 2018 to 2022 remained higher at 21.65/100,000 compared to the national average of 13.64/100,000 among students from 2004 to 2021 [19]. Moreover, with over 15,000 reported cases in 2022 and localized incidence rates exceeding 100/100,000 in some areas [18], the overall PTB situation in Chongqing remains concerning. Therefore, Chongqing should implement more effective identification and management measures for patients with PTB and latent infections to further reduce the risk of PTB among students.

Analyzing the epidemiological characteristics of student PTB, we found a male-to-female ratio of 1.24:1, consistent with previous studies [9]. This suggests that while there is a difference in risk between male and female students, the disparity is not substantial. The slightly higher incidence among males may be attributed to their larger activity range and more frequent interactions with peers, whereas females generally have a smaller activity range and place greater emphasis on personal hygiene. The minimal difference in risk between genders may be related to the relatively enclosed environment of schools and limited infection pathways [43]. Regarding grade or age distribution, the majority of cases occurred among high school students (13‐18 years), accounting for 67.03%, followed by college students (≥19 years) at 29.45%, while primary school students (7‐12 years) and kindergarten students (3‐6 years) constituted a smaller proportion. High school and college students exhibited higher PTB risks compared to younger students, possibly due to waning effectiveness of Bacillus Calmette-Guérin vaccine protection over time as reported as reported elsewhere [44]. Previous meta-analyses indicated that the risks of latent tuberculosis infection among students in China, attributed to crowded school environments compared to settings in Europe and the United States [9]. Analysis of detection methods and time to diagnosis revealed that passive discovery, such as referral and follow-up, accounted for 69.66% of PTB cases, with over half of patients with PTB (47.54%) experiencing a delay of more than 14 days from symptom onset to seeking medical attention. Moreover, the increasing proportion of positive etiological results warrants attention due to heightened transmission risks associated with these cases. Moreover,the proportion of patients with positive etiological results has shown an upward trend, consistent with previous studies on student TB in Chongqing [41]. This rise is likely due to the increased availability of molecular diagnostic equipment in Chongqing and a revision in the diagnostic criteria for strong positivity from “induration diameter ≥20 mm” to “≥15 mm.” Although the increase in positive cases is attributed to these objective factors, caution is warranted, as studies indicate that patients with positive etiological results have a higher bacterial load and greater transmission risk [45]. These findings underscore the importance of routine preventive measures such as morning and afternoon health checks and monitoring absenteeism due to illness to facilitate timely case detection and management [46]. Treatment success rates exceeding 90% among students may be attributed to milder health impacts and higher treatment compliance compared to other demographic groups [47,48]. Analysis of case reporting trends for student PTB cases in Chongqing reveals that in 2016, the peak reporting month was March, coinciding with physical examinations for high school and college entrance exams. In 2017, peaks were observed in both March and December, likely due to the inclusion of December as a physical examination period before exams. The September peak in 2018 is attributed to the integration of PTB screening into school entry examinations starting that year [41]. Since 2018, reporting peaks have consistently occurred in March, September, and December, aligning with patterns observed in other regions of China [43,49]. This indicates that large-scale health screenings associated with major exams and school entry are crucial for identifying student PTB cases.

Using map visualization, trend surface analysis, and spatial autocorrelation analysis, this study analyzed the geographical distribution of student PTB in Chongqing, revealing significant spatial clustering of student PTB. Global spatial autocorrelation analysis consistently indicated Moran *I* values of >0 across all years except 2018, signifying significant spatial clustering of PTB cases throughout the region. Local spatial autocorrelation analysis identified high-high clusters in northeastern regions, including Kaizhou, Yunyang, Wushan, Wuxi, and southeastern areas, including Pengshui, Qianjiang, and Wulong, consistent with incidence rate maps and spatial trend surface analysis results. Previous studies have identified these areas as high-risk regions for PTB among the general population in Chongqing [50,51]. The higher total number of patients and increased prevalence of latent infections elevate the infection risk among student populations in these areas, attributable to several factors. First, these regions are predominantly rural and mountainous with lower economic development and limited health care services. Moreover, residents, mostly farmers and ethnic minorities, may lack awareness of PTB and adequate self-protection measures, compounded by limited access to health care services, resulting in delayed diagnosis and ongoing transmission [40]. Thus, targeted prevention and control measures in these high-risk areas are essential, necessitating policy backing and enhanced funding. Moreover, LISA maps indicate that in 2019, the southern region was a high-high cluster, while the northern region showed no clustering. However, from 2020 to 2021, this clustering pattern changed, with the south becoming nonclustered and the north emerging as a high-high cluster. This change is unlikely to be significantly influenced by the COVID-19 pandemic, as pandemic control measures such as mask-wearing, movement restrictions, and school closures [38] would generally reduce incidence in both regions. Analysis of incidence data from 2019 to 2021 suggests that the change to no clustering in the south is likely related to a decrease in case numbers. Conversely, the northern region’s shift to high-high clustering is not due to an increase in incidence, in fact the overall trend of case numbers in the area for 2019‐2021 is decreasing, which may have occurred because the incidence in the north are still at a higher levels.

Spatiotemporal clustering analysis highlighted likely and secondary clusters of student PTB concentrated in northeastern and southeastern Chongqing, overlapping with hot spot areas identified in local spatial autocorrelation analysis. Accordingly, future efforts should not only advance overall prevention and control measures in these regions but also implement specific measures to reduce student PTB incidence within schools. For instance, studies recommend incorporating γ-interferon release tests during medical examinations for students in high-incidence areas to effectively screen for latent infections [52,53]. Clustering areas were also identified in the central urban areas of Yuzhong District and northern Hechuan District, possibly influenced by high population density and significant population mobility [51]. Furthermore, clustering of student PTB in Chongqing was concentrated between 2017 and 2020, indicating some improvement in recent years. However, due to persistent high-risk areas for PTB in Chongqing and the rapid transmission rate among students, vigilance in student PTB prevention and control efforts remains essential.

### Limitations

This study has several limitations. First, student PTB incidence data were derived from the TBIMS within the CISDP, potentially underestimating actual levels of PTB incidence by excluding undetected and untreated patients. Second, inconsistencies in the geographic information of townships (the smallest administrative units) registered in the TBIMS prevented a more granular assessment of PTB spatiotemporal characteristics at the township level. Third, numerous factors contribute to the spatiotemporal clustering of PTB, including economic conditions, health care service levels, and the allocation of prevention and control funding, and the extent to which these factors influence regional disease incidence levels requires further investigation.

### Conclusions

Our findings indicate a gradual decline in student PTB incidence rates in Chongqing, Southwest China, albeit at persistently elevated levels. High school and college students account for the majority of PTB cases among students, predominantly detected passively, with high proportions experiencing delays in diagnosis and positive etiological results. While COVID-19 control measures have had some impact on reducing incidence levels, the primary factor appears to be the implementation of tuberculosis screening measures during student health examinations. Spatial trend surface analysis and spatial autocorrelation analysis underscored strong spatial clustering of student PTB, with high-high clusters persisting in northeastern and southeastern Chongqing. Spatiotemporal scan analysis highlighted high case reporting within clustered areas, posing elevated risks of epidemic spread within these regions. Therefore, sustained efforts are necessary to enhance comprehensive prevention and control measures across high-risk regions of Chongqing to reduce student PTB incidence. Proactive screening strategies should be prioritized to minimize underreporting of student cases and identify carriers of latent infections.

## Supplementary material

10.2196/64286 Multimedia Appendix 1 Supplemental materials containing further data on the geographic locations of counties in Chongqing, Southwest China; the incidence of pulmonary tuberculosis (PTB) in Chongqing, Southwest China; and the epidemiological characteristics of students with PTB in Chongqing, Southwest China.

## Abbreviations

CISDP: Chinese Information System for Disease Control and Prevention
LISA: local indicators of spatial autocorrelation
LLR: log likelihood ratio
PTB: pulmonary tuberculosis
RR: relative risk
TBIMS: Tuberculosis Information Management System
WHO: World Health Organization
