# Supplementary material for: Epidemiological Characteristics and Spatiotemporal Clustering of Pulmonary Tuberculosis Among Students in Southwest China From 2016 to 2022: Analysis of Population-Based Surveillance Data
Source: JMIR Public Health Surveill. 2024 Sep 24;10:e64286. doi: 10.2196/64286 (PMC11462631; doi:10.2196/64286)
Supplement: Multimedia Appendix 1 [file publichealth-v10-e64286-s001.docx]

**Supplemental Materials**

**Table S1** Geographic locations of counties in Chongqing, Southwest China

| **Location** | **Latitude** | **longitude** |
| --- | --- | --- |
| Banan | 29.4056 | 106.5367 |
| Beibei | 29.8083 | 106.3922 |
| Bishan | 29.5949 | 106.2234 |
| Dadukou | 29.4903 | 106.4888 |
| Dazhu | 29.4888 | 105.7765 |
| Fuling | 29.7056 | 107.3852 |
| Hechuan | 29.9755 | 106.2730 |
| Jiangbei | 29.6125 | 106.5803 |
| Jiangjin | 29.2930 | 106.2556 |
| Jiulongpo | 29.5081 | 106.5170 |
| Kaizhou | 31.1630 | 108.3885 |
| Liangping | 30.6763 | 107.7979 |
| Nanan | 29.5068 | 106.6507 |
| Nanchuan | 29.1606 | 107.0945 |
| Qijiang | 28.9463 | 106.7108 |
| Qianjiang | 29.5360 | 108.7666 |
| Rongchang | 29.4078 | 105.5911 |
| Shapingba | 29.5472 | 106.4634 |
| Tongliang | 29.8473 | 106.0525 |
| Tongnan | 30.1933 | 105.8359 |
| Wanzhou | 30.8102 | 108.4041 |
| Wulong | 29.3282 | 107.7555 |
| Yongchuan | 29.3590 | 105.9236 |
| Yubei | 29.7208 | 106.6267 |
| Yuzhong | 29.5589 | 106.5754 |
| Changshou | 29.8603 | 107.0766 |
| Chengkou | 31.9499 | 108.6600 |
| Dianjiang | 30.3293 | 107.3306 |
| Fengdu | 29.8662 | 107.7264 |
| Fengjie | 31.0206 | 109.4590 |
| Pengshui | 29.2965 | 108.1611 |
| Shizhu | 30.0021 | 108.1096 |
| Wushan | 31.0770 | 109.8740 |
| Wuxi | 31.4009 | 109.5654 |
| Xiushan | 28.4517 | 109.0027 |
| Youyang | 28.8444 | 108.7635 |
| Yunyang | 30.9330 | 108.6926 |
| Zhongxian | 30.3028 | 108.0331 |

**Table S2** Incidence of PTB in total Population and students Tuberculosis in Chongqing ,southwest China, 2016–2022

| **Years** | **Total population** | | | **Students** | | | |
| --- | --- | --- | --- | --- | --- | --- | --- |
|  | **Number of population**  **（10,000）** | **Number of case** | **Incidence rate**  **（/ 1000,000）** | **Number of students enrolled（10,000）** | **Number of cases** | **Incidence rate**  **（/ 1000,000）** | **% student among all PTB case** |
| 2016 | 3048.43 | 20199 | 66.26 | 540.43 | 1617 | 29.92 | 8.01 |
| 2017 | 3075.16 | 18619 | 60.55 | 547.42 | 1624 | 29.67 | 8.72 |
| 2018 | 3101.79 | 17744 | 57.21 | 556.11 | 1835 | 33.00 | 10.34 |
| 2019 | 3124.32 | 16737 | 53.57 | 571.06 | 1533 | 26.84 | 9.16 |
| 2020 | 3208.93 | 10063 | 31.36 | 583.42 | 1393 | 23.88 | 13.84 |
| 2021 | 3212.43 | 13456 | 41.89 | 592.59 | 1097 | 18.51 | 8.15 |
| 2022 | 3213.34 | 11185 | 34.81 | 594.23 | 821 | 13.82 | 7.34 |
| Total | 21984.40 | 108003 | 49.38 | 3985.27 | 9920 | 24.89 | 9.18 |

**Table S3** Epidemiological Characteristics of students with PTB in Chongqing ,southwest China, 2016–2022

| **Variables** | **Total**  **(n=108003)** | **2016**  **（n=1617）** | **2017（n=1624）** | **2018（n=1835）** | **2019（n=1533）** | **2020（n=1393）** | **2021（n=1097）** | **2022**  **（n=821）** |
| --- | --- | --- | --- | --- | --- | --- | --- | --- |
| **Gender** |  |  |  |  |  |  |  |  |
| Male | 5484 | 913 | 933 | 1042 | 824 | 761 | 598 | 413 |
| Female | 4436 | 704 | 691 | 793 | 709 | 632 | 499 | 408 |
| **Grade/Age (years)** |  |  |  |  |  |  |  |  |
| Kindergarten students(3-6) | 4 | 0 | 1 | 1 | 1 | 1 | 0 | 0 |
| Primary school students(7-12) | 346 | 57 | 45 | 58 | 48 | 63 | 40 | 35 |
| High school students (13-18) | 6649 | 994 | 1086 | 1253 | 1058 | 954 | 744 | 560 |
| College school students (≥19) | 2921 | 566 | 492 | 523 | 426 | 375 | 313 | 226 |
| **Interval between first symptoms occurrence to hospital visit (days)** |  |  |  |  |  |  |  |  |
| 0-14 | 5204 | 858 | 897 | 942 | 801 | 710 | 554 | 442 |
| 15-29 | 1750 | 262 | 267 | 304 | 285 | 275 | 215 | 142 |
| 30-44 | 1262 | 213 | 198 | 247 | 204 | 162 | 138 | 100 |
| 45-59 | 404 | 61 | 53 | 88 | 63 | 62 | 47 | 30 |
| ≥60 | 1300 | 223 | 209 | 254 | 180 | 184 | 143 | 107 |
| **Ways of discovery** |  |  |  |  |  |  |  |  |
| Clinical consultation | 1970 | 363 | 371 | 347 | 308 | 308 | 195 | 78 |
| Recommended for symptoms | 140 | 11 | 10 | 36 | 21 | 32 | 19 | 11 |
| Referral | 5106 | 638 | 737 | 834 | 826 | 809 | 678 | 584 |
| Following up | 1804 | 441 | 375 | 436 | 202 | 132 | 118 | 100 |
| Physical examinations | 828 | 151 | 107 | 165 | 159 | 112 | 86 | 48 |
| Others | 72 | 13 | 24 | 17 | 17 | 0 | 1 | 0 |
| **Aetiological results** |  |  |  |  |  |  |  |  |
| Positive | 2748 | 212 | 211 | 439 | 481 | 562 | 455 | 388 |
| Negative | 7062 | 1392 | 1386 | 1355 | 1034 | 825 | 640 | 430 |
| No results | 110 | 13 | 27 | 41 | 18 | 6 | 2 | 3 |
| **Treatment outcomes** |  |  |  |  |  |  |  |  |
| Cured | 2006 | 182 | 166 | 262 | 344 | 410 | 366 | 276 |
| Treatment completed | 7440 | 1375 | 1375 | 1491 | 1142 | 924 | 695 | 438 |
| Default | 15 | 4 | 1 | 3 | 2 | 5 | 0 | 0 |
| Death | 8 | 0 | 1 | 3 | 0 | 1 | 1 | 2 |
| Failure | 38 | 8 | 2 | 7 | 3 | 12 | 3 | 3 |
| Transferred to multi-drug resistance treatment | 131 | 14 | 7 | 16 | 13 | 17 | 25 | 39 |
| Others | 282 | 34 | 72 | 53 | 29 | 24 | 7 | 63 |

**Table S4** The monthly incidence of student PTB in Chongqing ,southwest China, 2016-2022.

| **Months** | **2016** | **2017** | **2018** | **2019** | **2020** | **2021** |
| --- | --- | --- | --- | --- | --- | --- |
| January | 87 | 58 | 227 | 181 | 111 | 94 |
| February | 77 | 102 | 96 | 74 | 43 | 64 |
| March | 313 | 281 | 145 | 91 | 71 | 90 |
| April | 179 | 122 | 131 | 127 | 102 | 86 |
| May | 124 | 114 | 147 | 115 | 134 | 75 |
| June | 110 | 118 | 123 | 92 | 136 | 68 |
| July | 100 | 86 | 114 | 102 | 99 | 76 |
| August | 120 | 91 | 137 | 121 | 126 | 94 |
| September | 124 | 137 | 201 | 192 | 170 | 116 |
| October | 131 | 113 | 144 | 149 | 153 | 122 |
| November | 126 | 140 | 138 | 106 | 110 | 78 |
| December | 126 | 262 | 232 | 183 | 138 | 134 |
